# True Angina Pectoris Immediately After Cervical Disc Herniation Surgery for Preoperative Cervical Angina Symptoms: A Case Report

**DOI:** 10.7759/cureus.28313

**Published:** 2022-08-23

**Authors:** Takashi Abe, Takafumi Tanei, Yusuke Nishimura, Ryuta Saito

**Affiliations:** 1 Neurosurgery, Nagoya University Hospital, Nagoya, JPN

**Keywords:** chest pain, cervical disc herniation, anterior cervical discectomy and fusion, angina pectoris, cervical angina

## Abstract

Cervical angina, originating from cervical spine disorders, is a rare clinical syndrome presenting with chest pain mimicking angina pectoris. A rare case of cervical angina with cervical disc herniation requiring coronary artery stenting because of a true angina pectoris attack immediately after cervical spine surgery is reported. A 77-year-old woman presented with persistent pain around the neck, bilateral chest, left shoulder, and left back. She also complained of numbness and motor weakness in her left upper limb. Cervical spine imaging examinations showed instability at C4-5 and a calcified herniated disc with spinal cord compression at C4-6. She had a history of hypertension, diabetes mellitus not requiring insulin, and atrial fibrillation for which she was on anticoagulant therapy. The preoperative electrocardiogram and transthoracic echocardiography were within normal limits, and thus further cardiac study was considered unnecessary by the cardiologist. The anterior cervical discectomy and fusion were safely performed. However, she suddenly started to complain of left anterior chest pain with a cold sweat one hour after the surgery. An emergency electrocardiogram showed ischemic changes, and coronary angiography showed 99% stenosis at the right main coronary artery. A coronary stent was placed and good dilatation was achieved. Preoperative cervical angina symptoms such as numbness, motor weakness, and pain were improved immediately after surgery. The left chest pain also disappeared completely after coronary artery stent placement. Postoperative cervical imaging assessments showed good spinal decompression. She was discharged home without neurological deficits and no cardiac symptoms.

## Introduction

Cervical angina, originating from cervical spine (C-spine) disorders, is a rare clinical syndrome presenting with anterior chest pain or retrosternal pain mimicking angina pectoris [1-3]. The nature of cervical angina, commonly exacerbated by cervical motion or movement of the upper extremity, is either continuous for more than 30 minutes or paroxysmal, lasting less than five seconds, and described as a sharp or deep ache, tightness, or crushing. The pain is usually accompanied by neck pain, upper arm radicular symptoms, and occipital headaches. In addition, autonomic and sympathetic signs including dyspnea, nausea, dizziness, or diplopia coexist in more than half of patients. The diagnosis of cervical angina is often delayed or underdiagnosed because there are no diagnostic criteria. C-spine X-rays and magnetic resonance imaging (MRI) are critical for establishing a diagnosis of cervical angina [4]. A definitive diagnosis of cervical angina can only be made once coronary artery disease is ruled out because the symptoms of both closely resemble each other. Therefore, assessments of coronary artery disease, such as cardiac enzymes on blood testing, electrocardiogram (ECG), coronary computed tomography (CT) angiography, or coronary angiography should be performed for highly suspicious cases [4-8].

A rare case of cervical angina with cervical disc herniation requiring coronary artery stenting because of a true angina pectoris attack immediately after C-spine surgery is described.

## Case presentation

**Figure 1:**
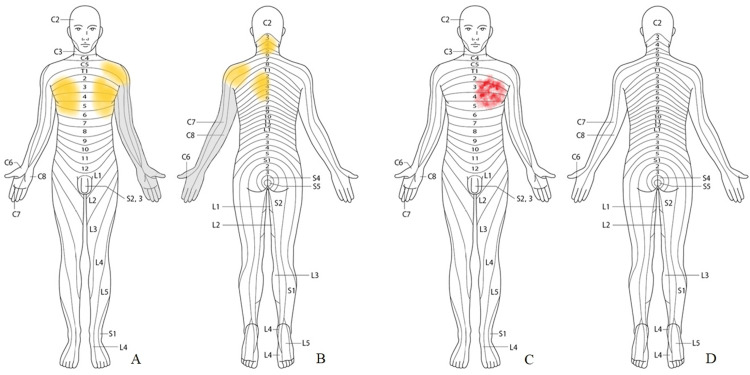
Schematic diagrams showing the location of pain A, B: Preoperative symptoms of persistent pain are located around the neck, bilateral chest, left shoulder, and left back (colored yellow). Numbness is located in her left arm and all fingers (colored gray). C, D: The true angina pectoris attack immediately after the cervical surgery presents as left chest pain with cold sweat (colored red).

A 77-year-old woman presented with persistent pain around the neck, left shoulder, and left subscapular area, as well as bilateral anterior chest pain, more prominent on the left side (Figures 1A, 1B). She also complained of numbness in her left arm and all fingers. Moderate true muscle weakness not due to pain, scored as grade 4 muscle strength on a manual muscle test, was detected in her left upper extremity including deltoid, biceps, triceps, and wrist muscles. Although the deep tendon reflexes of the upper extremities, ankle jerk, and plantar reflexes were normal, bilateral patellar tendon reflexes were hyperactive.

**Figure 2:**
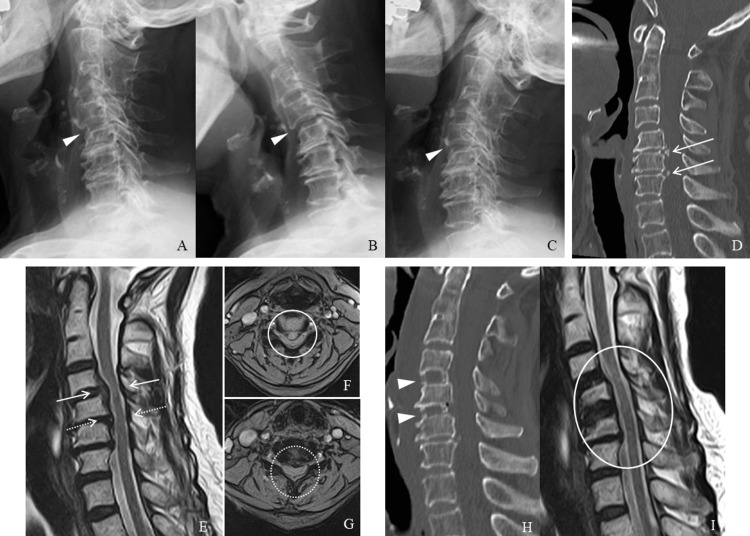
Pre- and postoperative cervical imaging examinations A-G: Preoperative cervical imaging examinations. A-C: Lateral views of the cervical spine X-ray showing instability at C4-5 (arrowhead). D: Computed tomography shows mild posterior longitudinal ligament ossification at the C4-6 levels (arrows). E: Magnetic resonance image, sagittal view, shows cervical disc herniation at C4-6 levels (arrows: C4/5, dotted arrows: C5/6). F, G: Magnetic resonance image, axial views, demonstrating high compression of the spinal cord (F: circle, C4/5, G: dotted circle, C5/6). H, I: Postoperative cervical imaging examinations showing good cage positions (arrows) and spinal decompression (circle) (H: computed tomography; I: magnetic resonance image).

**Figure 3:**
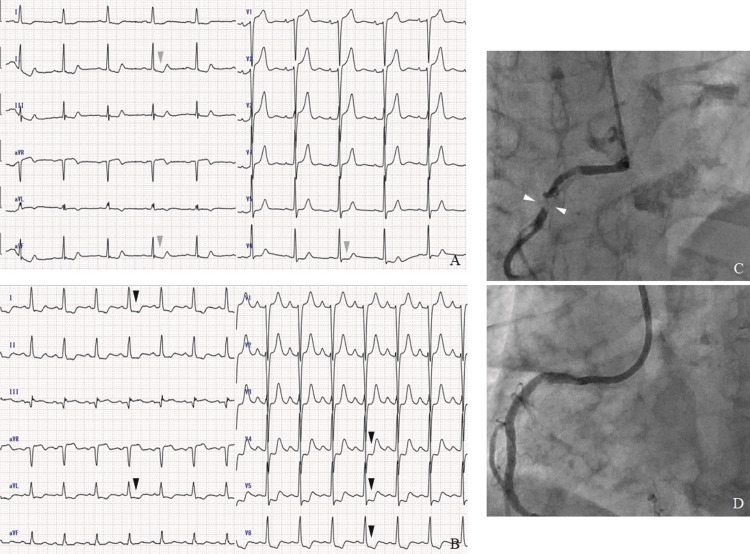
Pre- and postoperative cardiac examinations A: Preoperative electrocardiogram shows mild ST changes (gray arrowheads). B: Emergency electrocardiogram shows ST depression in V4-6, I, and aVL (black arrowheads). C: Emergency coronary angiography detects 99% stenosis at the right main coronary artery (arrowheads: 99% stenosis). D: After placement of a coronary stent, coronary angiography shows good dilatation of the right coronary artery.

Lateral views of the C-spine X-ray showed spinal instability at the C4-5 level (Figures 2A-2C). CT and MRI of the C-spine demonstrated a calcified herniated disc with spinal cord compression at the C4-6 levels (Figures 2D-2G). She had a history of hypertension, diabetes mellitus not requiring insulin, and atrial fibrillation on anticoagulant therapy. The oral anticoagulant was discontinued from the day before surgery. She had no history of an apparent ischemic heart attack or heart failure, and the previous coronary CT angiography performed one year earlier showed mild calcification in the proximal part of the left main coronary artery. The preoperative ECG showed mild ST changes, unchanged from three months earlier (Figure 3A). Preoperative transthoracic echocardiography showed sinus rhythm, with mild left atrial and ventricular hypertrophy with a normal ejection fraction. Further cardiac study was considered unnecessary by the cardiologist because all of these findings fell within normal ranges.

The patient underwent anterior cervical discectomy and fusion at C4/5 and C5/6. The posterior longitudinal ligament was removed, and titanium-coated interbody fusion cages were inserted at C4/5 and C5/6. The surgical procedure was performed safely, with no intraoperative surgical complications. Surgical time was two hours and 33 minutes, and hemorrhage volume was 35 ml. Intraoperative vital signs and hemodynamics were stable throughout the procedure. The patient emerged uneventfully from the general anesthesia. After returning to the intensive care unit, she suddenly started to complain of left anterior chest pain with cold sweats one hour after surgery (Figures 1C, 1D). Emergency ECG showed ST depression in V4-6, I, and aVL (Figure 3B). Emergency coronary angiography showed 99% stenosis at the right main coronary artery (Figure 3C). Thus, a coronary artery stent was successfully placed at the right coronary artery with good arterial dilatation (Figure 3D). Preoperative symptoms of left shoulder pain and numbness of the left arm, pain of the left subscapular area, and right anterior chest pain, as well as motor weakness, improved immediately after surgery. The left chest pain also disappeared completely after coronary artery stent placement. Postoperative C-spine imaging assessments showed good spinal decompression and cage positions (Figures 2H, 2I). She was discharged home about two weeks after the surgery without neurological deficits and cardiac symptoms and was followed up for about one year.

## Discussion

Cervical angina was first recognized in patients with cervical spondylosis exhibiting paroxysmal chest pain without cardiac abnormalities in 1927 [9]. The term “cervical angina” was proposed as a clinical syndrome in the 1950s [10]. The prevalence of cervical angina has not been clearly established. Ozgur et al. reported atypical chest pain or subscapular pain in 14.9% of 241 patients who underwent C6/7 anterior cervical discectomy and fusion [11]. Sussman et al. found six patients with cervical angina among 44 patients (13.6%) presenting with atypical chest pain [4]. Chien et al. reported that the prevalence of cervical angina was 9.8% in a retrospective review of 1,655 patients with cervical spondylosis, neck pain, and associated symptoms [12]. Retrospective analysis showed that the chest pain of the present case was probably a mix of cervical angina and true angina from coronary artery disease.

The causative pathologies of cervical angina include herniated disc, ossification of the posterior longitudinal ligament, spinal cord infarction, lipomatosis, and discitis [1-3]. Cervical angina is mainly caused by cervical radiculopathy from the C4 to C8 roots innervating sensation of the anterior chest wall. The most responsible nerve is the C6 root (37%), followed by C7 (30%), C5 (27%), and C4 (4%) [4,13]. Cervical angina can also be induced by myelopathy, as in the present case [4,14]. Accordingly, various mechanisms of cervical angina have been proposed. Disruption of the following nervous system pathways, including the cervical sympathetic nervous system afferent to the heart and coronary arteries, the vertebral sinus nerve innervating spinal meninges, posterior longitudinal ligaments, intervertebral discs, and periosteum of the vertebras, or the ascending cardiac spinothalamic tract located in the posterior horn of the spinal cord, are reportedly associated with the development of cervical angina [15,16].

Conservative treatments for cervical angina consist of medication, collar immobilization, and physical therapy. An adequate trial of conservative treatment should be given because most cases of cervical angina can be successfully cured by conservative treatment [12,16,17]. Preoperative differentiation may be possible if sufficient time for diagnosis is taken before surgery. Surgical treatment is recommended if more than three months of conservative treatment fails with the presence of significant nerve root compression [4,12,13,17,18]. However, in cases of progressive myelopathy, as in the present case, surgical treatment must be considered promptly, and particular attention should be paid to the presence of coronary artery disease.

There are several guidelines for the perioperative management of coronary artery disease [5-8]. The Revised Cardiac Risk Index is the most widely used scoring system for risk assessment of perioperative cardiac events [7]. The index scores 1 or 0 points in each of the following six categories: (1) ischemic heart disease, (2) history of heart failure, (3) history of cerebrovascular disease, (4) diabetes mellitus requiring insulin, (5) renal dysfunction, and (6) high-risk surgery. The risk of perioperative cardiac events is classified according to the sum of all scores as low risk (0-1), intermediate risk (2), and high risk (3-6). The present case, with a total score of 0 points, was classified as low risk. Invasive coronary examinations are not recommended for low-risk patients [8]. On the other hand, the European Society of Cardiology guideline also predicts the possible risk of obstructive coronary artery disease by calculating three parameters: age, sex, and type of chest symptoms [6]. The risk group is classified as low risk (<5%), moderate risk (5-15%), or high risk (>15%). The score of the present case was 10% (female, 70 years older, non-anginal), falling within the moderate risk group.

In the guideline, the moderate and high-risk patients with multiple comorbidities are recommended to perform additional preoperative cardiac functional examinations such as stress ECG, stress echocardiography, dobutamine stress test, myocardial perfusion single photon emission computed tomography, stress myocardial perfusion MRI, and stress myocardial perfusion positron emission tomography [5-8]. These examinations can help in predicting the cardiac response to surgical stress.

## Conclusions

Most of the preoperative chest pain in the present case was considered to be cervical angina because it was persistent and not a typical ischemic attack. On the other hand, the postoperative chest pain was a typical ischemic attack with localized left chest pain and a cold sweat. The patient had previously been found to have mild calcification at the left main coronary artery, although the ischemic attack occurred on the opposite side. Therefore, the ischemic attack might have been induced by various operative factors, such as the invasive surgical procedure, intraoperative hemodynamic stress, and anesthetic drugs. Detailed preoperative coronary artery assessment of possible cervical angina may be necessary for elderly patients with risk factors for coronary artery disease, such as hypertension, diabetes mellitus, atrial fibrillation, and ventricular hypertrophy, even without a previous ischemic cardiac attack.
